# Waist Circumference and Cardiovascular Risk Profiles after Autologous Breast Reconstruction: A Nationwide Population-Based Cohort Study

**DOI:** 10.3390/jcm12083040

**Published:** 2023-04-21

**Authors:** Jeong Yeop Ryu, Myeong Jae Kang, Hyun Geun Cho, Jung Dug Yang, Joon Seok Lee

**Affiliations:** Department of Plastic and Reconstructive Surgery, School of Medicine, Kyungpook National University, Kyungpook National University Chilgok Hospital 807, Hoguk-ro, Buk-gu, Daegu 41404, Republic of Korea

**Keywords:** autologous breast reconstruction, abdominoplasty, waist circumference, cardiovascular risk profile

## Abstract

To date, few studies have examined changes in waist circumference and cardiovascular risk profile (CVRP) after autologous breast reconstruction. Therefore, this study aimed to investigate the effect of flap surgery using autologous tissue on waist circumference and CVRP through a nationwide population-based cohort study. In total, 6926 patients who underwent autologous breast reconstruction between 2015 and 2019 were considered. Of them, we evaluated 3444 patients who underwent the complete Korean National Health Insurance Service Health Screening (NHIS-HealS) before and after surgery. Body measurements, including waist circumference, weight, and body mass index; and CVRP, including blood pressure, fasting blood glucose, and cholesterol levels, were analyzed by type of surgery up to 3–4 years postoperatively. The body measurements of patients who underwent abdominal-based breast reconstruction were reduced 1–2 years after surgery, but returned to preoperative values 3–4 years after surgery. Regardless of the type of surgery, CVRP was worsened at both 1–2 years and 3–4 years after surgery, except for low-density lipoprotein values. Autologous breast reconstruction did not ameliorate the deterioration of CVRP over time. In addition, the abdominoplasty effect of abdominal-based breast reconstruction disappeared 1–2 years after surgery.

## 1. Introduction

Breast cancer is the most common cancer among women in the United States and affects over 4 million of women, often at a relatively young age [1]. With the increase in early screening and the development of modern medical technology, the mortality rate of breast cancer has significantly decreased. Accordingly, there has been a growing interest in life quality after breast cancer surgery, and the frequency of breast reconstruction has been increasing. Compared with women who receive mastectomy alone, women who also receive breast reconstruction are less affected by psychological distress and achieve more satisfaction with surgery outcomes [2].

There are two major methods for breast reconstruction after breast cancer surgery: namely, implant-based breast reconstruction and autologous breast reconstruction. Recent studies have shown that women who underwent autologous breast reconstruction were more satisfied, had lower complication rates, and enjoyed a better quality of life compared with women who underwent alloplastic breast reconstruction [3,4]. Therefore, there is a preference for autologous breast reconstruction among patients and breast reconstruction surgeons. Among the techniques for autologous breast reconstruction, the latissimus dorsi (LD) muscle or transversus rectus abdominis musculocutaneous (TRAM) and deep inferior epigastric perforator (DIEP) flaps in the abdomen can be used. Abdomen-based autologous breast reconstruction has the effect of abdominoplasty, as a large amount of subcutaneous fat tissue is also excised. In the clinic, the expectations for abdominoplasty are high among patients, which is one of the main factors in favor of the abdomen-based breast reconstruction method compared to the latissimus dorsi muscle method. However, few studies have examined whether the abdominoplasty effect is maintained in the longer term. According to our clinical experience, long-term follow-up after abdominal-based breast reconstruction often showed recurrence in obesity cases.

Moreover, there is controversy as to whether the removal of subcutaneous rather than visceral fat leads to changes in metabolic conditions. Klein et al. reported that abdominal liposuction did not improve the metabolic abnormalities associated with obesity and the cardiovascular risk profile associated with heart disease [5]. Swanson reported that triglyceride levels were significantly reduced in patients after liposuction or abdominoplasty [6]. 

This is the first nationwide population-based study to evaluate the effect of abdominoplasty based on body measurements such as weight, body mass index (BMI), and waist circumference and cardiovascular risk profiles (CVRPs) of patients who underwent abdominal-based autologous breast reconstruction.

## 2. Materials and Methods

### 2.1. Data Source

This nationwide study was based on the Korean National Health Insurance (NHI) Sharing Service (NHISS) database. More than 50 million Koreans are set to join the government-run NHI system, and approximately 97% of Koreans are currently enrolled in the NHI system [7]. The NHI database contains comprehensive patient data, including patient sociodemographic characteristics, all hospitalization and outpatient records, prescriptions for medications, prescriptions for surgery, fees, and diagnostic codes [8]. The diagnostic code is based on the Korean Standard Classification of Disease [modified version of the International Classification of Diseases, 10th edition (ICD-10)].

The National Health Insurance Service Health Screening (NHIS-HealS) program offers a two-stage screening test for Korean citizens [9]. In the first stage, screening is conducted every 1–2 years for all individuals in Korea, through surveys including medical interviews and lifestyle questionnaires, physical examinations, regular blood and urine tests, chest X-ray examinations, medical history, and smoking/drinking history. In the second stage, more detailed screening tests, such as endoscopy and ultrasonography, are performed according to age. The NHIS-HealS database gathers all data from the first-stage screening [9].

### 2.2. Study Population

#### 2.2.1. Cohort for Autologous Breast Reconstruction

NHI database patients who were diagnosed with breast cancer between January 2015 and December 2019 and received breast reconstruction using autologous tissue after total mastectomy were considered in this study. First, breast cancer patients were selected using the ICD code C50. Then, using the surgical fee, patients undergoing autologous breast reconstruction were identified as having received LD flaps for the N7140–N7142 operating codes, TRAM flaps for the N7143–N7146 operating codes, or DIEP flaps for the N7147 operating codes.

A one-year washout period from January to December 2014 was applied to identify patients who underwent further autologous breast reconstruction. 

#### 2.2.2. Cardiovascular Risk Profile

The CVRP included body measurements such as waist circumference, BMI, weight, blood pressure (BP), and fasting blood glucose (FBS). In addition, lipid profiles with total cholesterol, triglyceride (TG), and low-density lipoprotein cholesterol (LDL-C) levels were also included [10]. The preoperative baseline CVRP was verified in all patients who received NHIS-HealS and autologous breast reconstruction. If a patient had undergone NHIS-HealS more than twice before surgery, the most recent NHIS-HealS data were considered as baseline CVRP.

#### 2.2.3. Diagnostic Accuracy

Two plastic surgeons reviewed the medical records of all the patients who underwent autologous breast reconstruction from 2015 to 2019 in a single medical center to check the accuracy of the surgical codes based on sensitivity and specificity.

Each surgical code sensitivity was defined as the proportion of patients who received the N7140–N7142 operating codes among the patients who received LD flaps, the proportion of patients who received the N7143–N7146 operating codes among the patients who received TRAM flaps, or the proportion of patients who received the N7147 operating code among the patients who received DIEP flaps. In addition, the proportion of patients who did not receive LD flaps among the patients who did not receive a N7140–N7142 surgical code, the proportion of patients who did not receive TRAM flaps among the patients who did not receive a N7143–N7146 surgical code, and the percentage of patients who did not receive DIEP flaps among the patients who did not receive a N7147 surgical code were used to define each code specificity. 

A total of 316 patients were analyzed. The sensitivity of the code for the LD flap was 95.37% and the specificity was 100%. The sensitivity of the code for the TRAM flap was 100% and the specificity was 93.02%. The sensitivity and specificity of the code for the DIEP flap were both 100%.

### 2.3. Statistical Analysis

Body measurements and CVRP were evaluated preoperatively and 1–2 years and 3–4 years after surgery in patients who underwent breast reconstruction using abdominal-based flaps. CVRP is a continuous variable, and baseline CVRPs before surgery and 1–2 years or 3–4 years after surgery were compared for each autologous breast reconstruction surgery method. In addition, a regression analysis was performed to examine if each surgical method could be a factor affecting body measurement and CVRPs. All statistical analyses were performed using STATA Stata/MP2 (version 13.0; StataCorp, College Station, TX, USA). Statistical significance was set at *p* < 0.05.

## 3. Results

A total of 7454 patients in the NHI database received autologous breast reconstruction during the 2014–2019 period. After applying the washout period, 6926 patients remained during the 2015–2019 period. Among them, 2125 patients received LD flaps; 2624 patients received TRAM flaps; 2136 patients received DIEP flaps; 25 patients received both LD and TRAM flaps; 3 patients received both TRAM and DIEP flaps; and 13 patients received both LD and DIEP flaps (Table 1).

**Figure 1:**
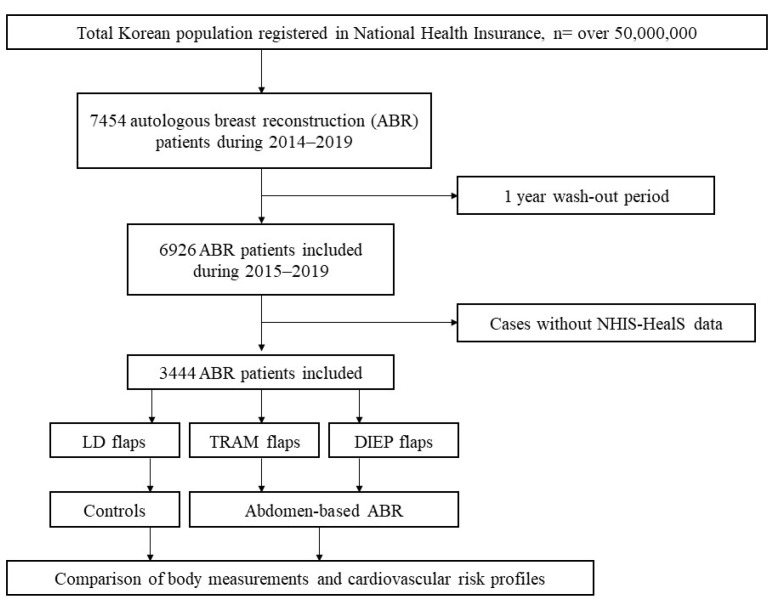
Flowchart of the study. ABR, autologous breast reconstruction; DIEP, deep inferior epigastric perforator; LD, latissimus dorsi; TRAM, transversus rectus abdominis musculocutaneous; NHIS-HealS, National Health Insurance Service Health Screening.

Among a total of 6926 patients, data for 3444 patients were extracted with both NHIS-HealS before surgery, 1–2 years after surgery, and NHIS-HealS 3–4 years after surgery (Figure 1).

### 3.1. Body Measurements 

Table 2 shows the waist circumference, weight, and BMI before and after surgery by surgical technique. First, there was no statistically significant difference in waist circumference before and 1–2 years after surgery among the patients who wore LD flaps. On the other hand, the patients who received TRAM flaps and DIEP flaps showed a statistically significant average decrease of 0.41 cm and 0.47 cm in waist circumference, respectively, 1–2 years after surgery. However, 3–4 years after surgery, no difference in waist circumference was observed before and after surgery for all patients. In particular, the waist circumference in patients who received TRAM and DIEP flaps returned to the preoperative 75 cm range 3–4 years after surgery. 

Second, there was no statistically significant difference in body weight before and 1–2 years after surgery for patients who received LD flaps. On the other hand, the patients who received TRAM and DIEP flaps had a statistically significant decrease in average body weight of 0.18 kg and 0.35 kg, respectively, 1–2 years after surgery. However, 3–4 years after surgery, no difference was observed in body weight before and after surgery for all patients. In particular, the body weight in patients who received TRAM and DIEP flaps returned to its preoperative value range 3–4 years after surgery.

Third, there was no difference in BMI for patients who received LD flaps before and 1–2 years after surgery. In patients who received TRAM flaps, the mean BMI decreased 1–2 years after surgery, but it was not statistically significant. Patients who received DIEP flaps had a statistically significant decrease in mean BMI 1–2 years after surgery. Three to four years after surgery, there was no statistically significant BMI change before and after surgery regardless of the surgical technique.

The regression analysis showed that, after 1–2 years, TRAM and DIEP flap surgeries were a statistically significant factor in reducing waist circumference, but the effect disappeared 3–4 years after surgery. TRAM and DIEP flap surgeries did not influence body weight and BMI in either postoperative period (Table 3).

### 3.2. Cardiovascular Risk Profile

Table 4 shows the cardiovascular risk profile without the lipid profiles before and after surgery by surgical technique. The systolic BP was increased 1–2 years and 3–4 years after surgery compared to before surgery in all patients. There was no statistically significant difference in diastolic BP in patients who received LD flaps 1–2 years after surgery, but a statistically significant increase was seen in patients who received TRAM and DIEP flaps. On the other hand, 3–4 years after surgery, the diastolic BP increased regardless of the type of surgery. Moreover, FBS, which is a measure of diabetes, was significantly increased 1–2 years and 3–4 years after surgery in all patients who underwent LD, TRAM, and DIEP flap surgery. In the regression analysis, neither TRAM nor DIEP flap surgery was a significant factor influencing systolic BP, diastolic BP, and FBS (Table 5).

Table 6 shows the lipid profiles before and after autologous breast reconstruction sorted by surgical technique. The mean value of total cholesterol increased with LD, TRAM, and DIEP flaps 1–2 years after surgery, but it was statistically significant only for the DIEP flap, and there was no significant increase 3–4 years after surgery. The triglyceride level significantly increased in all patients 1–2 years and 3–4 years after surgery. While there was no statistically significant difference 1–2 years after surgery, the LDL-C values in LD and TRAM flap surgery patients were significantly decreased 3–4 years after surgery compared to before surgery. In the regression analysis, neither TRAM nor DIEP flap surgery was a statistically significant factor affecting total cholesterol, triglyceride, and LDL-C levels. However, age was a significant factor influencing total cholesterol and triglyceride levels (Table 7).

## 4. Discussion

Several previous studies have examined the effect of removing subcutaneous fat tissue on metabolism. Klein et al. reported that liposuction had no significant effects on other risk factors for coronary heart disease, including blood pressure, fasting plasma glucose, insulin, and lipid concentrations, and concentrations of plasma markers of inflammation and insulin resistance (C-reactive protein, tumor necrosis factor-α, interleukin-6, and adiponectin) 10 to 12 weeks after liposuction [5]. However, Esposito et al. reported that various body measurements and total cholesterol and triglyceride levels decreased after 6 months of follow-up in a study conducted in 45 patients after large-volume liposuction [11]. Giugliano et al. also argued that liposuction was a safe approach free of metabolic sequelae in obese women; moreover, liposuction was associated with amelioration of insulin resistance and reduced circulating markers of vascular inflammation, possibly helping obese subjects to reduce their cardiovascular risk [12]. However, Bassetto et al. refuted the assertion by Giugliano et al. that these metabolic marker changes are caused by surgical maneuvers and stress. They argued that only insulin resistance showed positive changes [13]. In a prospective study of metabolic profiles after abdominal liposuction in 20 healthy volunteers, abdominal liposuction was found to significantly improve weight, BMI, and waist circumference 4 months after surgery by 4.6%, 4.6%, and 5.9%, respectively [14]. There were significant decrements in free fatty acid, glycerol, very-low-density lipoprotein cholesterol, and triglyceride levels. On the other hand, Mohammed et al. reported that CVRPs obtained from 10 to 208 weeks after removal of abdominal subcutaneous adipose tissue remained unchanged from baseline. These data demonstrated that removal of a large amount of abdominal subcutaneous adipose tissue by using liposuction does not improve CVRPs associated with abdominal obesity, despite a long-term reduction in body fat [15]. Thus, there is controversy about whether subcutaneous fat removal has a positive effect on metabolism, with no nationwide study conducted so far. 

Here, we compared patients who underwent autologous breast reconstruction with abdominal-based flaps with those who received LD flaps as controls through a nationwide study. In terms of waist circumference, patients who received TRAM and DIEP flaps had a statistically significant decrease in waist circumference 1–2 years after surgery. This demonstrated that abdominal-based autologous breast reconstruction was effective for abdominoplasty effects 1–2 years after surgery. However, 3–4 years after surgery, the waist circumference of patients who received TRAM and DIEP flaps returned to their preoperative values, suggesting that the abdominoplasty effects of abdominal-based breast reconstruction were not long-lasting. The patients who received LD flaps as a control group did not show a statistically significant change in waist circumference both 1–2 years and 3–4 years after surgery. The weight of patients who received TRAM and DIEP flaps was also decreased significantly 1–2 years after surgery, but returned to its preoperative values 3–4 years after surgery. 

Blood pressure was increased 1–2 years and 3–4 years after surgery in all patients regardless of whether they received LD, TRAM, and DIEP flaps, suggesting that blood pressure was not related to the type of autologous breast reconstruction. Fasting blood glucose was also increased 1–2 years and 3–4 years after surgery in all patients regardless of whether they received LD, TRAM, and DIEP flaps. The regression analysis confirmed that the different surgical methods in autologous breast reconstruction were not factors affecting blood pressure and FBS. 

As for the lipid profiles, total cholesterol and triglyceride levels were higher after surgery than before surgery, while LDL-C levels were lower. In particular, triglyceride levels increased significantly 1–2 years and 3–4 years after surgery. However, the regression analysis confirmed that the TRAM and DIEP flaps, which remove large amounts of adipose tissue, cannot be a factor affecting lipid profiles. It can be inferred that the triglyceride and total cholesterol levels may gradually increase with patient age. According to statistics on the general population in Korea, the prescribed amount of statin, which accounts for 90% of all the drugs used for the treatment of hypercholesterolemia, is increasing rapidly in Korea [16]. This may explain why LDL-C levels decrease over time in most patients who underwent autologous breast reconstruction. 

Overall, our study suggests that abdominal-based autologous breast reconstruction is not a factor influencing the metabolic profile, and that the abdominoplasty effect in reducing waist circumference lasted only 1–2 years. Abdominal-based autologous breast reconstruction is a good option for breast reconstruction. However, many plastic surgeons choose abdominal-based autologous breast reconstruction with TRAM or DIEP flaps because they believe that it has abdominoplasty effects. In addition, they expect the removal of large amounts of adipose tissue to affect the metabolic profile, which has not yet been definitively demonstrated. Our nationwide study suggests that the evidence on the abdominoplasty and metabolic effects of abdominal-based autologous breast reconstruction is not sufficient to determine a benefit of abdominal-based autologous breast reconstruction. If all that is aspired to is a decrease in waist circumference lasting 1–2 years, the evidence suggests choosing abdominal-based autologous breast reconstruction over other surgical methods.

This study has several limitations. Since the diagnoses were made based on the ICD-10 codes, detailed diagnostic classifications reflecting breast cancer severity (i.e., stage of breast cancer and amount of breast tissue removed) were not available. Medical images, photographs, and radiologic findings were also not included in this database. Thus, bias in the diagnostic classifications may exist. However, we took care to validate the diagnostic accuracy of the ICD-10 codes. Moreover, the Korean NHI is a reasonably accurate database. The insurance review teams of each general hospital in Korea verify the ICD-10 code and surgical fees before hospitals claim medical fees. Afterwards, the Health Insurance Review & Assessment, which is another national public institution separate from the NHI, performs reverification. Therefore, the bias for misdiagnosis in the present study is low. Another limitation is that our analyses included only Korean participants; therefore, the results might not be generalizable to people of other ethnicities. There are better criteria for CVRP instead of waist circumference, such as waist-to-hip ratio, but we aimed to measure the effectiveness of abdominoplasty in terms of plastic surgery with waist circumference. Additionally, the hip circumference or hip ratio were not included on the national health examination list in Korea, which is for general health indicators of the whole nation. Additionally, there were no data on type of breast cancer, medical complications, postoperative death or recurrence, fat distribution in upper and lower body parts, and visceral fat measurement in the database. Additionally, there can be differences in morphology and adipogenic capacity between pre- and post-menopausal women. However, there was no information on whether the participants had a national health examination before or after menopause. Bias can occur depending on these factors and we will consider this in further study. Additionally, in this study, we wanted to discuss how the surgical method of autologous breast reconstruction affects the fat distribution. It would be better to compare all factors affecting the fat distributions but it would be practically difficult. Thus, we extracted the control group randomly among the patients with same autologous breast reconstruction. Since this is big data analysis, if the data were randomly extracted, the ratio of menopausal women in the patient group and the ratio of menopausal women in the control group would be almost the same. There would be other strong CV risk modifiers, such as radiotherapy and chemotherapeutics; however, there would be no difference between cancer stages, which determines the use of radiotherapy and chemotherapeutics between abdominal-based reconstruction and other reconstructions.

This study also has several strengths. First, to the best of our knowledge, this is the first comparative study to investigate body measurements, CVRPs, and lipid profiles in patients with autologous breast reconstruction nationwide. Importantly, a nationwide study provides not only a large number of participants, but also a minimized selection bias. Finally, because all Korean NHI claims data are publicly available and can be studied by any researchers worldwide, our study has high data transparency.

## 5. Conclusions

In this nationwide study, abdominal-based autologous breast reconstruction, which removes large amounts of adipose tissue, did not improve patients’ metabolic profile. In addition, since the abdominoplasty effect of reducing waist circumference, which is expected by many patients and plastic surgeons alike, did not last more than 1–2 years, it cannot serve as evidence for choosing abdominal-based autologous breast reconstruction over other methods.

## Figures and Tables

**Table 1:** Patients with autologous breast reconstruction during 2015–2019 in South Korea.

Age and Flap	LD	TRAM	DIEP	LD + TRAM	TRAM + DIEP	LD + DIEP	Total
10–19	2	0	0	0	0	0	2
20–29	29	10	9	0	0	0	48
30–39	322	224	216	4	1	0	767
40–49	990	1124	961	13	0	9	3097
50–59	629	1011	773	6	2	4	2425
60–69	132	231	173	2	0	0	538
>70	21	24	4	0	0	0	49
total	2125	2624	2136	25	3	13	6926

LD, latissimus dorsi; TRAM, transversus rectus abdominis muscle; DIEP, deep inferior epigastric perforator.

**Table 2:** Comparison of body measurements before and after surgery sorted by surgical techniques.

			1–2 Years Follow-Up				3–4 Years Follow-Up			
	Operative Techniques		Obs	Mean	95% CIs	*p*-Value	Obs	Mean	95% CIs	*p*-Value
Waist circumference (cm)	LD	Preoperative	959	75.12	74.60–75.65	0.1695	992	75.15	74.64–75.66	0.0506
		Postoperative	959	75.39	74.88–75.91		992	75.56	75.03–76.08	
	TRAM	Preoperative	1158	75.17	74.80–75.53	0.0189 *	1313	75.55	75.18–75.92	0.9413
		Postoperative	1158	74.76	74.43–75.09		1313	75.56	75.20–75.92	
	DIEP	Preoperative	996	75.19	74.76–75.61	0.0159 *	1041	75.32	74.89–75.76	0.6967
		Postoperative	996	74.72	74.36–75.08		1041	75.25	74.85–75.65	
	LD + TRAM	Preoperative	17	75.26	71.59–78.94	0.6997	14	75.07	70.77–79.38	0.6894
		Postoperative	17	74.76	71.18–78.35		14	75.71	71.93–79.50	
	TRAM + DIEP	Preoperative	1	73			1	73		
		Postoperative	1	77			1	77		
	LD + DIEP	Preoperative	7	71.01	67.45–74.58	0.9349	0	No obs	No obs	No obs
		Postoperative	7	70.83	67.52–74.14		0	No obs	No obs	
Weight (kg)	LD	Preoperative	970	57.74	57.20–58.29	0.5599	1016	57.69	57.16–58.22	0.5146
		Postoperative	970	57.67	57.16–58.19		1016	57.77	57.25–58.29	
	TRAM	Preoperative	1178	56.42	56.10–56.74	0.0449 *	1339	57.02	56.67–57.37	0.7481
		Postoperative	1178	56.24	55.93–56.54		1339	57.05	56.71–57.39	
	DIEP	Preoperative	1013	57.69	57.27–58.11	0.0022 **	1067	57.84	57.42–58.26	0.5863
		Postoperative	1013	57.34	56.93–57.74		1067	57.78	57.35–58.20	
	LD + TRAM	Preoperative	17	57.27	52.69–61.85	0.6704	14	58.11	52.74–63.48	0.7802
		Postoperative	17	57.48	53.05–61.91		14	57.95	52.83–63.07	
	TRAM + DIEP	Preoperative	1	54			1	54		
		Postoperative	1	54.3			1	54.3		
	LD + DIEP	Preoperative	0	No obs	No obs	No obs	7	54.01	46.63–61.40	0.0852
		Postoperative	0	No obs	No obs		7	56.71	52.15–61.28	
BMI (kg/m^2^)	LD	Preoperative	970	22.84	22.63–23.04	0.7816	1016	22.81	22.61–23.01	0.0759
		Postoperative	970	22.85	22.65–23.05		1016	22.9	22.70–23.10	
	TRAM	Preoperative	1178	22.76	22.63–22.86	0.2256	1339	22.94	22.81–23.07	0.1226
		Postoperative	1178	22.71	22.60–22.84		1339	23	22.87–23.13	
	DIEP	Preoperative	1013	22.91	22.76–23.07	0.0086 **	1067	22.98	22.83–23.14	0.7027
		Postoperative	1013	22.79	22.65–22.94		1067	23	22.85–23.16	
	LD + TRAM	Preoperative	17	22.62	21.01–24.23	0.3779	14	22.48	20.70–24.28	0.7
		Postoperative	17	22.81	21.13–24.49		14	22.58	20.77–24.39	
	TRAM + DIEP	Preoperative	1	24			1	24		
		Postoperative	1	23.4			1	23.4		
	LD + DIEP	Preoperative	7	21.67	19.76–23.58	0.3059	7	21.67	19.76–23.59	0.0565

LD, latissimus dorsi; TRAM, transversus rectus abdominis muscle; DIEP, deep inferior epigastric perforator; CIs, confidence intervals. * *p* < 0.05, ** *p* < 0.01.

**Table 3:** Linear regression for body measurements before and after surgery sorted by multiple variables.

		1Linear Regression				3Linear Regression			
		Coefficient	Std. Err.	*p*-Value	95% CIs	Coefficient	Std. Err.	*p*-Value	95% CIs
Waist circumference	Age	0.0000	0.0002	0.819	0.0000–0.0000	−0.0001	0.0002	0.609	−0.00050.0000
	Year of surgery	−0.0028	0.0011	0.012	−0.0049	−0.0044	0.0012	0	−0.0067
	Type of surgery								
	LD	Reference				Reference			
	TRAM	−0.0087	0.0035	0.013 *	−0.0156	−0.005	0.0035	0.152	−0.0118
	DIEP	−0.0082	0.0036	0.024 *	−0.0153	−0.0046	0.0037	0.205	−0.0118
	LD + TRAM	−0.0105	0.0194	0.589	−0.0486	0.0052	0.0221	0.813	−0.0380
	LD + DIEP	−0.0086	0.0301	0.776	−0.0677	0.0471	0.0311	0.131	−0.0140
Weight	Age	−0.0002	0.0001	0.291	−0.0004	−0.0006	0.0002	0.000 ***	−0.0009 *
	Year of surgery	−0.0005	0.0008	0.546	−0.0021	−0.0026	0.0009	0.005 **	−0.0043
	Type of surgery								
	LD	Reference				Reference			
	TRAM	−0.0021	0.0025	0.398	−0.0071	0.0000	0.0027	0.987	−0.0054
	DIEP	−0.0049	0.0026	0.063	−0.0100–0.0003	−0.0018	0.0029	0.534	−0.0074–0.0003
	LD + TRAM	0.0031	0.0142	0.825	−0.0248–0.0003	−0.0056	0.0176	0.752	−0.0400–0.0003
	LD + DIEP	0.0307	0.0221	0.164	−0.0125–0.0003	0.0524	0.0248	0.035 *	0.00385–0.000
BMI	Age	0.0000	0.0001	0.968	−0.0003–0.0003	−0.0004	0.0002	0.006 **	−0.0007–0.0003
	Year of surgery	−0.0005	0.0008	0.508	−0.0021	−0.003	0.0009	0.001 **	−0.0049
	Type of surgery								
	LD	Reference				Reference			
	TRAM	−0.003	0.0026	0.245	−0.0080	−0.0011	0.0028	0.098	−0.0065–0.0044
	DIEP	−0.0058	0.0027	0.028 *	−0.0111–0.0044r	−0.0024	0.0029	0.414	−0.0081–0.0044
	LD + TRAM	0.0053	0.0144	0.714	−0.0229–0.0044	−0.0015	0.0178	0.933	−0.0363–0.0044
	LD + DIEP	0.0312	0.0223	0.162	−0.0126–0.0044	−0.0554	0.025	0.027 *	0.00634–0.004

LD, latissimus dorsi; TRAM, transversus rectus abdominis muscle; DIEP, deep inferior epigastric artery perforator; BMI, body mass index; err, error; CIs, confidence intervals. * *p* < 0.05, ** *p* < 0.01, *** *p* < 0.001.

**Table 4:** Comparison of cardiovascular risk profiles without lipid profiles before and after surgery sorted by surgical techniques.

			1–2 Years Follow-Up				3–4 Years Follow-Up			
	Operative Techniques		Obs	Mean	95% CIs	*p*-Value	Obs	Mean	95% CIs	*p*-Value
SBP	LD	Preoperative	970	116.61	115.72–117.49	0.0004 ***	1016	116.39	115.54–117.24	0.0000 ***
		Postoperative	970	118.21	117.29–119.13		1016	118.89	118.01–119.79	
	TRAM	Preoperative	1177	116.55	115.80–117.29	0.0003 ***	1339	116.77	116.07–117.47	0.0000 ***
		Postoperative	1177	117.96	117.17–118.75		1339	118.76	117.99–119.54	
	DIEP	Preoperative	1013	116.64	115.82–117.45	0.0012 **	1067	116.65	115.84–117.46	0.0000 ***
		Postoperative	1013	118.04	117.20–118.88		1067	118.48	117.62–119.33	
	LD + TRAM	Preoperative	17	115.76	107.32–124.21	0.1709	14	114.43	106.01–122.84	0.158
		Postoperative	17	123.94	111.58–136.30		14	120	110.42–129.58	
	TRAM + DIEP	Preoperative	1	110			1	110		
		Postoperative	1	130			1	130		
	LD + DIEP	Preoperative	7	114.29	102.57–126.01	0.1052	7	114.29	102.57–126.01	0.2094
		Postoperative	7	121.71	109.56–133.86		7	120.86	104.08–137.64	
DBP	LD	Preoperative	970	73.19	72.57–73.81	0.2164	1016	72.97	72.37–73.57	0.0002 ***
		Postoperative	970	73.59	72.98–74.21		1016	74.21	73.60–74.83	
	TRAM	Preoperative	1177	73.12	72.60–73.65	0.0033 **	1339	73.2	72.71–73.69	0.0034 **
		Postoperative	1177	73.96	73.42–74.50		1339	74	73.48–74.52	
	DIEP	Preoperative	1013	73.16	72.57–73.76	0.0052 **	1067	73.23	72.65–73.80	0.0052 **
		Postoperative	1013	74.05	73.44–74.66		1067	74.12	73.53–74.71	
	LD + TRAM	Preoperative	17	77.06	73.74–80.38	0.4864	14	76.36	72.84–79.88	0.3986
		Postoperative	17	79	73.62–84.38		14	78.43	73.66–83.20	
	TRAM + DIEP	Preoperative	1	70			1	70		
		Postoperative	1	77			1	77		
	LD + DIEP	Preoperative	7	70.43	63.19–77.67	0.6407	7	70.43	63.19–77.67	0.0941
		Postoperative	7	71.43	62.08–80.78		7	77.57	66.57–88.58	
FBS (mg/dL)	LD	Preoperative	970	94.96	93.87–96.04	0.0224 *	1016	95.17	94.09–96.25	0.0028 **
		Postoperative	970	96.13	95.08–97.17		1016	96.7	95.66–97.94	
	TRAM	Preoperative	1177	93.79	92.98–94.60	0.0009 ***	1339	94.34	93.46–95.21	0.0000 ***
		Postoperative	1177	95.34	94.40–96.28		1339	96.56	95.64–97.48	
	DIEP	Preoperative	1013	94.07	93.17–94.97	0.0104 *	1066	94.11	93.18–95.05	0.0001 ***
		Postoperative	1013	95.11	94.18–96.04		1066	95.81	94.84–96.78	
	LD + TRAM	Preoperative	17	93.53	87.56–99.50	0.502	14	93.71	86.44–100.99	0.6991
		Postoperative	17	95.94	88.45–103.44		14	95.14	89.07–101.22	
	TRAM + DIEP	Preoperative	1	104			1	104		
		Postoperative	1	101			1	101		
	LD + DIEP	Preoperative	7	97.14	82.67–111.61	0.1765	7	97.14	82.67–111.61	0.1291
		Postoperative	7	89.43	84.15–94.71		7	88.14	81.95–94.33	

LD, latissimus dorsi; TRAM, transversus rectus abdominis muscle; DIEP, deep inferior epigastric perforator; SBP, systolic blood pressure; DBP, diastolic blood pressure; FBS, fasting blood sugar; CIs, confidence intervals. * *p* < 0.05, ** *p* < 0.01, *** *p* < 0.001.

**Table 5:** Linear regression for cardiovascular risk profiles without lipid profiles before and after surgery sorted by multiple variables.

		1Linear Regression				3Linear Regression			
		Coefficient	Std. Err.	*p*-Value	95% CIs	Coefficient	Std. Err.	*p*-Value	95% CIs
Systolic BP	Age	0.0003	0.0003	0.288	−0.0003–0.0009	0.0002	0.0003	0.534	−0.0004–0.0008
	Year of Surgery	0.0003	0.0016	0.877	−0.0030–−0.0035	−0.0005	0.0018	0.789	−0.0039–0.0030
	Types of surgery								
	LD	Reference				Reference			
	TRAM	−0.0031	0.0052	0.552	−0.0133–0.0071	−0.0057	0.0053	0.281	−0.0160–0.0046
	DIEP	−0.0024	0.0054	0.654	−0.0130–0.0082	−0.0069	0.0055	0.215	−0.0178–0.0040
	LD + TRAM	0.0579	0.0292	0.048 *	0.0006–0.1152	0.0245	0.0338	0.469	−0.0418–0.0907
	LD + DIEP	0.0488	0.0453	0.282	−0.0401–0.1377	0.0284	0.0476	0.551	−0.0649–0.1218
Diastolic BP	Age	−0.0007	0.0003	0.047 *	−0.0014–0.0000	−0.0011	0.0003	0.002 **	−0.0017–−0.0004
	Year of Surgery	−0.0001	0.0019	0.952	−0.0038–0.0036	−0.0007	0.0020	0.724	−0.0046–0.0032
	Types of surgery								
	LD	Reference				Reference			
	TRAM	0.0061	0.0060	0.305	−0.0056–0.0179	−0.0054	0.0060	0.369	−0.0170–0.0063
	DIEP	0.0063	0.0062	0.311	−0.0059–0.0184	−0.0042	0.0063	0.503	−0.0165–0.0081
	LD + TRAM	0.0135	030335	0.688	−0.0523–0.0792	0.0021	0.0382	0.955	−0.0729–0.0771
	LD + DIEP	−0.0027	0.0520	0.959	−0.1046–0.0993	0.0745	0.0539	0.167	−0.0311–0.1802
FBS	Age	0.0006	0.0004	0.126	−0.0002–0.0013	0.0007	0.0004	0.041 *	0.0000–0.0014
	Year of Surgery	0.0046	0.0020	0.020*	0.0007–0.0085	−0.0020	0.0021	0.327	−0.0061–0.0020
	Types of surgery								
	LD	Reference				Reference			
	TRAM	−0.0002	0.0063	0.0977	−0.0125–0.0122	0.0046	0.0062	0.458	−0.0076–0.0168
	DIEP	−0.0074	0.0065	0.0254	−0.0202–0.0053	−0.0012	0.0065	0.852	−0.0140–0.0116
	LD + TRAM	0.0086	0.0353	0.0807	−0.0605–0.0778	0.0019	0.0399	0.963	−0.0764–0.0802
	LD + DIEP	−0.0844	0.0547	0.123	−0.1916–0.0228	−0.1055	0.0563	0.061	−0.2158–0.0049

BP, blood pressure; FBS, fasting blood sugar; LD, latissimus dorsi; TRAM, transversus rectus abdominis muscle; DIEP, deep inferior epigastric artery perforator; err, error; CIs, confidence intervals. * *p* < 0.05, ** *p* < 0.01.

**Table 6:** Comparison of lipid profiles before and after surgery sorted by surgical technique.

			1–2 Years Follow-Up				3–4 Years Follow-Up			
	Operative Techniques		Obs	Mean	95% CIs	*p*-Value	Obs	Mean	95% CIs	*p*-Value
Total chol (mg/dL)	LD	Preoperative	741	195.21	192.69–197.74	0.153	739	194.09	191.55–196.64	0.4996
		Postoperative	741	197.04	194.40–199.67		739	195	192.38–197.63	
	TRAM	Preoperative	894	197.36	194.99–199.74	0.1104	935	199.18	196.84–201.53	0.574
		Postoperative	894	199.15	196.71–201.60		935	198.5	196.08–200.91	
	DIEP	Preoperative	683	194.23	191.66–196.81	0.0153 *	688	195.48	192.84–198.13	0.1947
		Postoperative	683	197.53	194.80–200.26		688	197.38	194.56–200.20	
	LD + TRAM	Preoperative	16	180	171.85–188.15	0.1729	12	181	171.31–190.69	0.1173
		Postoperative	16	191.94	169.87–214.00		12	198.08	170.44–225.73	
	TRAM + DIEP	Preoperative	1	167			1	167		
		Postoperative	1	175			1	175		
	LD + DIEP	Preoperative	6	196.33	157.74–234.92	0.8664	6	196.33	157.74–234.92	0.2841
		Postoperative	6	199.33	156.91–241.76		6	176.17	159.86–192.47	
TG (mg/dL)	LD	Preoperative	726	103.77	98.99–108.55	0.0000 ***	717	103.03	98.29–107.76	0.0000 ***
		Postoperative	726	117.71	110.64–124.78		717	119.44	112.65–126.23	
	TRAM	Preoperative	867	107.08	102.43–111.72	0.0000 ***	904	108.29	103.97–112.61	0.0000 ***
		Postoperative	867	117.62	112.68–122.55		904	119.75	114.85–124.65	
	DIEP	Preoperative	667	103.69	98.57–108.81	0.0001 ***	670	103.01	97.74–108.28	0.0000 ***
		Postoperative	667	116.57	110.46–122.67		670	117.31	111.69–122.93	
	LD + TRAM	Preoperative	16	89.06	62.96–115.17	0.0160*	12	92.25	59.31–125.19	0.1737
		Postoperative	16	131.87	87.39–176.36		12	119.83	62.87–176.79	
	TRAM + DIEP	Preoperative	1	51			1	51		
		Postoperative	1	84			1	84		
	LD + DIEP	Preoperative	6	65.33	44.82–85.85	0.7467	6	65.33	44.82–85.85	0.4526
		Postoperative	6	62.83	48.94–76.73		6	59.67	42.40–76.93	
LDL-C (mg/dL)	LD	Preoperative	721	113.85	111.56–116.13	0.4723	712	113.19	110.85–115.52	0.0264 *
		Postoperative	721	112.97	110.54–115.40		712	110.27	107.82–112.73	
	TRAM	Preoperative	864	117.76	114.25–121.28	0.0908	899	118.34	115.22–121.45	0.0022 **
		Postoperative	864	114.62	112.35–116.90		899	113.17	110.88–115.45	
	DIEP	Preoperative	662	113.34	110.96–115.72	0.7074	665	114.67	112.17–117.16	0.9409
		Postoperative	662	113.84	111.28–116.40		665	114.51	110.27–118.74	
	LD + TRAM	Preoperative	16	100.56	88.13–113.00	0.8501	12	99.67	84.78–114.55	0.3777
		Postoperative	16	99.06	79.37–118.76		12	107	85.03–128.97	
	TRAM + DIEP	Preoperative	1	93			1	93		
		Postoperative	1	99			1	99		
	LD + DIEP	Preoperative	6	123.17	87.55–158.78	0.8828	6	123.17	87.55–158.78	0.2399
		Postoperative	6	126	91.55–160.45		6	100.33	87.40–113.27	

LD, latissimus dorsi; TRAM, transversus rectus abdominis muscle; DIEP, deep inferior epigastric perforator; CIs, confidence intervals. * *p* < 0.05, ** *p* < 0.01, *** *p* < 0.001.

**Table 7:** Linear regression for lipid profiles before and after surgery by multiple variables.

		1Linear Regression f				3Linear Regression f			
		Coefficient	Std. Err.	*p*-Value	95% CIs	Coefficient	Std. Err.	*p*-Value	95% CIs
Total cholesterol	Age	−0.0006	0.0005	0.305	−0.0016–0.0005	−0.0014	0.0006	0.012 *	−0.0025–−0.0003
	Year of surgery	−0.0016	0.0032	0.609	−0.0078–0.0046	0.0001	0.0035	0.985	−0.0068–0.0069
	Type of surgery								
	LD	Reference				Reference			
	TRAM	−0.0005	0.0091	0.959	−0.0184–0.0174	−0.0062	0.0095	0.512	−0.0248–0.0124
	DIEP	0.0081	0.0097	0.405	−0.0110–0.0272	0.0056	0.0102	0.582	−0.0143–0.0256
	LD + TRAM	0.0371	0.0463	0.422	−0.0536–0.1278	0.0633	0.0558	0.257	−0.0461–0.1728
	LD + DIEP	0.0105	0.0750	0.889	−0.1365–0.1575	−0.0995	0.0786	0.874	−0.2536–0.0545
Triglyceride	Age	−0.0037	0.0023	0.116	−0.0082–0.0009	−0.0088	0.0024	0.000 ***	−0.0136–−0.0040
	Year of surgery	−0.0088	0.0134	0.513	−0.0351–0.0175	−0.0369	0.0150	0.0014 *	−0.0664–−0.0074
	Type of surgery								
	LD	Reference				Reference			
	TRAM	−0.0006	0.0388	0.988	−0.0767–0.0754	−0.0166	0.0410	0.685	−0.0970–0.0638
	DIEP	0.0303	0.0412	0.461	−0.0504–0.1111	0.0464	0.0439	0.291	−0.0397–0.1325
	LD + TRAM	0.2986	0.1938	0.123	−0.0814–0.6786	0.0596	0.2376	0.802	−0.4064–0.5255
	LD + DIEP	−0.2569	0.3141	0.414	−0.8728–0.3591	−0.3911	0.3343	0.242	−1.0467–0.2645
LDL	Age	0.0009	0.0011	0.443	−0.0013–0.0031	0.0001	0.0012	0.945	−0.0023–0.0025
	Year of surgery	0.0012	0.0065	0.859	−0.0116–0.0139	0.0054	0.0076	0.480	−0.0096–0.0203
	Type of surgery								
	LD	Reference				Reference			
	TRAM	−0.0017	0.0188	0.926	−0.0386–0.0351	−0.0075	0.0208	0.716	−0.0483–0.0332
	DIEP	0.0243	0.0120	0.225	−0.0150–0.0634	0.0296	0.0223	0.184	−0.0140–0.0732
	LD + TRAM	−0.0360	0.0936	0.700	−0.2196–0.1476	0.0632	0.1200	0.599	−0.1721–0.2985
	LD + DIEP	0.0810	0.1517	0.593	−0.2165–0.3785	−0.1295	0.1689	0.443	−0.4606–0.2016

LDL, low-density lipoprotein; LD, latissimus dorsi; TRAM, transversus rectus abdominis muscle; DIEP, deep inferior epigastric artery perforator; err, error; CIs, confidence intervals.. * *p* < 0.05, *** *p* < 0.001.

## Data Availability

Not applicable.
